# Rapid and Sensitive Detection of Thrombospondin-2 Using Nanoparticle Sensors for Cancer Screening and Prognosis

**DOI:** 10.3390/mi16030354

**Published:** 2025-03-20

**Authors:** Maziyar Kalateh Mohammadi, Seyedsina Mirjalili, Md Ashif Ikbal, Hao Xie, Chao Wang

**Affiliations:** 1 School of Electrical, Computer, and Energy Engineering, Arizona State University, Tempe, AZ 85287, USA; mkalateh@asu.edu (M.K.M.); smirjali@asu.edu (S.M.); ashifikbal051@gmail.com (M.A.I.); 2 Biodesign Center for Molecular Design and Biomimetics, Arizona State University, Tempe, AZ 85287, USA; 3 Department of Oncology, Mayo Clinic, Rochester, MN 55905, USA; xie.hao@mayo.edu

**Keywords:** cancer, screening, gold nanoparticles, thrombospondin-2, point of care (POC)

## Abstract

Thrombospondin-2 (THBS2) is a prevailing prognostic biomarker implicated in different cancer types, such as deadly colorectal, pancreas, and triple-negative breast cancers. While the current methods for cancer-relevant protein detection, such as enzyme-linked immunosorbent assay (ELISA), mass spectrometry, and immunohistochemistry, are feasible at advanced stages, they have shortcomings in sensitivity, specificity, and accessibility, particularly at low concentrations in complex biological fluids for early detection. Here, we propose and demonstrate a modular, in-solution assay design concept, Nanoparticle-Supported Rapid Electronic Detection (NasRED), as a versatile cancer screening and diagnostic platform. NasRED utilizes antibody-functionalized gold nanoparticles (AuNPs) to capture target proteins from a minute amount of sample (<10 µL) and achieve optimal performance with a short assay time by introducing active fluidic forces that act to promote biochemical reaction and accelerate signal transduction. This rapid (15 min) process serves to form AuNP clusters upon THBS2 binding and subsequently precipitate such clusters, resulting in color modulation of the test tubes that is dependent on the THBS2 concentration. Finally, a semiconductor-based, portable electronic device is used to digitize the optical signals for the sensitive detection of THBS2. High sensitivity (femtomolar level) and a large dynamic range (five orders of magnitude) are obtained to analyze THBS2 spiked in PBS, serum, whole blood, saliva, cerebrospinal fluids, and synovial fluids. High specificity is also preserved in differentiating THBS2 from other markers such as cancer antigen (CA) 19-9 and bovine serum albumin (BSA). This study highlights NasRED’s potential to enhance cancer prognosis and screening by offering a cost-effective, accessible, and minimally invasive solution.

## 1. Introduction

Thrombospondin-2 (THBS2) has emerged as a significant prognostic biomarker across a broad spectrum of cancers, including colorectal cancer (CRC) [1,2], gastric cancer (GC), colitis-associated colorectal cancer (CAC) [3,4,5], pancreatic ductal adenocarcinoma (PDAC) [6], ovarian cancer [7], triple-negative breast cancer (TNBC) [8], non-small-cell lung cancer (NSCLC) [9,10], oral cavity squamous cell carcinoma (OSCC) [11], cervical cancer [12], bladder cancer [13], hepatocellular carcinoma [14], and melanoma [15]. THBS2 plays a critical role in the remodeling of the tumor microenvironment, the promotion of tumor angiogenesis, and cancer cell migration and invasion. Therefore, there is a strong association between high levels of THBS2 and poor clinical outcomes [5,12,16]. Its relevance to a wide variety of diseases makes THBS2 a potential cornerstone biomarker for not only prognostic evaluation but also early cancer screening. Its detection in various biological fluids, including blood [6], plasma, serum, urine [13], and synovial fluid [17], enables its use as a minimally invasive biomarker for diagnostics and monitoring.

Conventional diagnostic technologies typically used to measure THBS2 include enzyme-linked immunosorbent assay (ELISA) [6,8], mass spectrometry [18], polymerase chain reaction (PCR) [19], and immunohistochemistry (IHC) [20]. However, achieving sufficient sensitivity and specificity using point-of-care (POC) platforms remains challenging, particularly at low levels in complex biological fluids. Furthermore, THBS2 detection in clinical settings needs to be differentiated from background biomarkers, for example, CA19-9 for PDAC [6], to ensure accuracy.

To meet the urgent need for high-accuracy POC cancer screening, this study introduces an in-solution, single-tube, modular assay platform specifically designed to deliver high sensitivity, specificity, and operational efficiency for biomarker analysis. Leveraging our success in developing nanosensors [21,22,23,24], we introduce nanoparticle-supported rapid electronic detection (NasRED) as a versatile and simple-to-use platform and demonstrate its applications in detecting THBS2 in various biological fluids. Briefly, upon the introduction of THBS2 in solution, antibody-functionalized gold nanoparticles (AuNPs), via biotin–streptavidin reaction, bind to the target and precipitate in clusters, with the cluster sizes and quantities correlating with the concentration of THBS2 in the solution. The physical precipitation process of AuNPs is accelerated by centrifugation, which acts to increase the AuNP and protein collision rate in solution and create a high-concentration region of the reagents at the bottom of the tube to favor detection at enhanced sensitivity. Afterward, vortex agitation is used following incubation to resuspend non-reacting AuNPs, thus preserving the specificity. The above sensing process essentially separates AuNP into clusters and free-floating monomers. Because the AuNP clusters increase with the THBS2 concentration, fewer AuNP monomers will be present in the supernatant, thus causing a decrease in the optical extinction. The optical signals are collected using a semiconductor-based portable electronic device (PED), digitalized using customized circuits that stabilize the readout signals, transmitted through Wi-Fi or Bluetooth modules, and then readily stored for analysis [22]. Using THBS2 detection as an example, NasRED demonstrates its feasibility as a novel platform that can make an impact in physician’s offices to transform cancer screening and prognostics.

## 2. Materials and Methods

### 2.1. Materials and Reagents

Recombinant Human Thrombospondin-2 Protein (1635-T2) and Human Thrombospondin-2 Biotinylated Antibody (BAF1635) were purchased from R&D Systems (Minneapolis, MN, USA). Human Pooled Serum (HPS) (C817B46), Single-Donor Human Whole Blood (WB) (IWB1K2E10ML), Single-Donor Human Saliva (IRHUSLS5ML), and Pooled Human Cerebrospinal Fluid (CSF) (IRHUCSF1ML) were purchased from Innovative Research Inc (Novi, MI, USA). Single Human Synovial Fluid (991-42-S-1) was purchased from Medix Biochemica (St. Louis, MO, USA). CA 19-9 antigen (DAGA-768) was purchased from Creative Diagnostics (Shirley, NY, USA). Bovine serum albumin (BSA) and molecular biology-grade glycerol from Sigma-Aldrich (St. Louis, MO, USA). Phosphate-buffered saline, 10× solution (10×PBS) and DNase/RNase-free distilled water used in the experiments were purchased from Fisher Scientific (Hampton, NH, USA). The 80 nm streptavidin-functionalized AuNPs were purchased from Cytodiagnostics Inc (Burlington, ON, Canada) and dispersed in 20% *v*/*v* glycerol and 1 wt% BSA buffer.

### 2.2. Selection of Optimal AuNP Size for Biosensing Probe Preparation

The size of the AuNPs significantly impacts their absorption wavelength, directly influencing the localized surface plasmon resonance (LSPR) phenomenon. Larger nanoparticles typically exhibit absorption peaks at longer wavelengths due to enhanced oscillations of conduction electrons on the particle surface. Conversely, smaller nanoparticles absorb light at shorter wavelengths [25]. Additionally, the number of binding sites on each particle decreases with the size of the nanoparticles. The AuNP optimization also takes into account the assay time, which is shortened for larger AuNP sizes. Based on our previous studies [24], 80 nm AuNPs achieved a large dynamic range, high sensitivity, and rapid signal transduction well.

### 2.3. Sensing Dilution Buffer Preparation

The 10× phosphate-buffered saline stock buffer was mixed with glycerol and BSA and deionized water to create a 1× PBS dilution buffer with a final concentration of 1× PBS, 20% *v*/*v* glycerol, and 1 wt% BSA. This dilution buffer (pH~7.4) was used to prepare AuNP sensors, antigen and antibody solutions, and diluted biological media.

### 2.4. AuNP Functionalization

The AuNPs in the sensing dilution buffer solutions were functionalized with anti-THBS2 antibodies using a biotin–streptavidin reaction. In detail, 50 μL of AuNPs (0.13 nM) was mixed with an excessive amount of 20 μL of antibodies (2 μM) in a 1.5 mL Eppendorf tube and incubated for 2 h at room temperature followed by 12 h at 4 °C. Then, the tube was gently vortexed, and 630 μL of sensing dilution buffer was added to the tube and centrifuged for 10 min at 10 krpm (9600× *g*) to precipitate. After that, the supernatant (650 μL) was removed and replaced with 650 μL of the sensing dilution buffer. The procedure was repeated 2 times to remove excessive antibodies. Finally, the concentration of coated AuNPs was adjusted to the desired optical density (OD) level (OD~0.5), or around 0.026 nM, using the sensing dilution buffer.

### 2.5. THBS2 Detection Experiments

First, 40 μL of WB and synovial fluid was mixed separately with 160 μL of sensing dilution buffer to make 20% of each matrix to minimize matrix-induced interferences. To prepare the target solutions, 5 μL of 4 μM THBS2 was diluted into different matrices individually (PBS, HPS, 20% WB, 20% synovial fluid, saliva, and CSF) to achieve final concentrations ranging from 10 nM to 100 fM. For detection, 18 μL of the prepared AuNP probes was mixed with 6 μL of THBS2 solutions at each concentration. As a negative control (NC), 6 μL of each biological matrix without THBS2 was mixed with the AuNP probe solutions, and the mixture went through the same sample processing steps to evaluate the matrix effect on the sensing solution. THBS2 sample tubes and the NC control tube were centrifuged at 3500 rpm, or 1180× *g*, incubated for 10 min, and vortexed for 5 s at 32.5 rps (1950 rpm).

### 2.6. Specificity in Different Matrices

To determine the specificity of the sensing probes, two other model proteins, e.g., Carbohydrate Antigen 19-9 (CA19-9) and BSA protein were chosen as a control. For this purpose, CA 19-9 and BSA were spiked and diluted into 100% HPS and saliva to reach the concentration of 10 nM. HPS and saliva solutions without THBS2 were used as a negative control, while THBS2 (10 nM) served as the positive control. Next, 6 μL of each sample was mixed with 18 μL functionalized AuNPs in a microcentrifuge tube and centrifuged at 1200× *g* and then incubated for 10 min followed by vortexing at 32.5 rps for 5 s.

### 2.7. Electronic Reader Design

The portable electronic detection (PED) system similar to our recent study [22] consisted of three essential components: (1) a light-emitting diode (LED; WP7113PGD, Kingbright, City of Industry, CA, USA), (2) a photodiode-integrated circuit (SEN-12787, SparkFun Electronics, Niwot, CO, USA), which incorporates a digital light sensor (APDS-9960, Broadcom, Palo Alto, CA, USA), and (3) a custom-fabricated tube holder created using a 3D printer (Qidi Tech X-Plus, Shanghai City, China) with black carbon fiber polycarbonate filament. The tube holder was specifically designed to securely accommodate standard 0.5 mL Labcon microcentrifuge tubes (Petaluma, CA, USA), ensuring precise alignment of the optical components. The photodiode APDS-9960 was operated with a bias voltage of 3.3 V and interfaced with a microcontroller (Atmega328) to process and digitize the signal output. This configuration enabled reliable electronic readouts of light intensity variations with minimal noise. All the electronic components, unless otherwise noted, were procured from DigiKey (Thief River Falls, MN, USA), and their integration was optimized for consistent performance in the detection setup.

### 2.8. Data Analysis

Our portable electronic reader measured the optical values of the tubes. Each tube was put in the tube holder, and its light transmission value was converted to a digital signal. For each individual tube, two values were recorded: one after mixing the AuNP sensors with target solutions (*T_1,i_*) and another one after completion of the sensing protocol (*T_2,i_*). These two sets of values were subtracted to remove background noises and obtain a delta transmission value (*T_t,i_*).

(1) $$T_{t,i}=T_{2,i}-T_{1,i}$$

Then, the tubes were positioned in 5 different orientations, and such calibrated signals *T_t,i_* were measured along each orientation and then averaged to account for tube variability (*T_t_*).

(2) $$T_{t}=\frac{\sum_{i}(T_{t,i})}{5},(i=1\;to\;5)$$

A baseline value for each matrix was measured by loading a tube with 18 μL of AuNP sensors and 6 μL of the media, representing the highest AuNP concentration and lowest signal intensity $T_{B}$ in the absence of THBS2. In addition, 24 μL of the media was loaded into the tube and measured, representing the highest possible signal intensity $T_{M}$ in a hypothetical total aggregation of AuNPs. Then, the measured transmission values were normalized as follows:

(3) $$S_{PED}=\frac{T_{t}-T_{B}}{T_{M}-T_{B}}$$

from 1 to 0, e.g., 1 representing no aggregation and no target, and 0 indicating all AuNPs precipitated at a high target concentration. The signals (*S_PED_*) were plotted into a graph and fitted using a biphasic dose–response equation in Origin 2024b software (OriginLab, Northampton, MA, USA) by orthogonal distance regression algorithm to consider the data and error weight in fitting calculations [22]. The fitting equation is as follows:

(4) $$S_{PED}(C)=S_{L}+({S_{H}-S}_{L})\left[\frac{p}{1+10^{({log}_{x01}-C)h_{1}}}+\frac{1-p}{1+10^{({log}_{x02}-C)h_{2}}}\right]$$

where *S_PED_* is the plotted signal; *C* is the logarithm of concentration; *S_L_* and *S_H_* are the lowest and highest signals; *x*_01_ and *x*_02_ are half maximal effective concentrations (EC_50_-1); and EC_50_-2, *h*_1_, and *h*_2_ are hill slopes for the first and second phases; and *p* is the steepness. Also, the error bars were calculated by the standard deviation of the five values of each tube following:

(5) $$\sigma =\frac{\sqrt{\sum {\left(T_{t,i}-T_{t}\right)}^{2}}}{n-1}$$

where *n* is 5 for the tube variations. The coefficient of variation (*CV*%) for each tube was calculated by the following:

(6) $$CV\%=\frac{\sigma }{T_{x}}\times 100\%$$

where *T_x_* is either *T*_1,*i*_, *T*_2,*i*_, or *T_t_*_,*i*_. The limit of detection (LoD) was defined as the lowest concentration of the target calculated using the modified formula [22,26]:

(7) $$S_{LoD}=S_{NC}-1.645\times \left(\sigma_{NC}+\sigma_{LC}\right)$$

where $\sigma_{LC}$ is the error from the sample with the lowest non-zero protein concentration. $S_{LoD}$ was then plotted against the corresponding target concentrations to determine the LoD.

## 3. Results and Discussion

### 3.1. Sensing Mechanism

**Figure 1:**
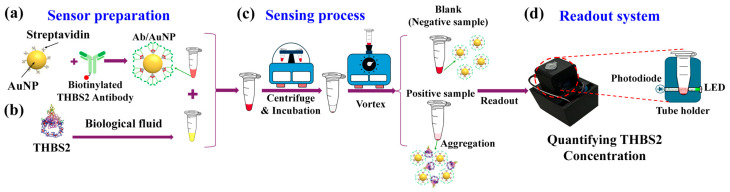
Design scheme for NasRED-based THBS2 detection. (**a**) Streptavidin-coated gold nanoparticles (AuNPs) were functionalized with anti-THBS2 antibodies to create specific probes targeting THBS2. (**b**) THBS2 was spiked into PBS and body fluids such as whole blood, serum, synovial fluid, and saliva. These samples (red tubes) were prepared through serial dilutions ranging from 10 nM to 100 fM, alongside a negative control containing the fluid matrix without THBS2 (yellow tube). When mixed, a biochemical binding reaction occurs between THBS2 and anti-THBS2 antibodies on the AuNP surface, leading to clustering. (**c**) Clustering was enhanced using active fluidic forces generated by centrifugation, which precipitates nanoparticles to the bottom of the tube, thereby locally enhancing reagent concentration. During incubation, clusters grow, while non-reacted nanoparticles are later resuspended using vortex agitation. (**d**) The colorimetric signals from the tubes were measured using a portable electronic device (PED). This device comprises a 3D-printed tube chamber, an LED, a photodiode, and a custom circuit board to digitize the analog signals from transmitted light through the tube.

Within the context of our sensing assay, streptavidin-coated AuNPs were functionalized with biotinylated THBS2 antibodies, forming multivalent sensing probes (Figure 1a). The biotin–streptavidin reaction is a strong and highly specific non-covalent interaction with a dissociation constant (Kd) on the order of ≈10^−14^ M and has a wide range of applications in affinity tagging, detection, and imaging. Biotinylated components can be precisely linked together through streptavidin bridges, enabling the creation of complex biosensors [27,28,29]. THBS2 samples were prepared by spiking in PBS and body fluids (Figure 1b) along with negative controls (NC; here, blank samples without THBS2). The prepared samples were then mixed with AuNP colloids, triggering antibody–antigen interactions and inducing AuNP aggregation in positive samples. The signal transduction was accelerated by the active fluidic force generated during centrifugation. Subsequently, vortex agitation redispersed the unbound AuNPs (Figure 1c). The transparency of the aggregated tubes would increase because of AuNP precipitation, which was quantified by our PED (Figure 1d).

### 3.2. Sensing Protocol Optimization

Unlike conventional assays, such as ELISA, which rely on passive diffusion and equilibrium conditions over long incubation times (often hours), NasRED operates in a quasi-equilibrium state. External controls, such as centrifugation, actively accelerate diffusion and reaction kinetics, bypassing the need for passive equilibrium. Vortex fluidic force helps ensure the elimination of non-specific interactions. Centrifugation and vortex agitation dynamically adjust the distribution of AuNPs and proteins in the sensing buffer solutions, creating controlled conditions that promote efficient interactions between the nanoparticles and target molecules. This unique approach enables NasRED to achieve rapid signal transduction and significantly shorter assay times while maintaining sufficient biochemical stability for reliable sensing.

Finding an optimal sensing condition is, therefore, necessary to achieve the best sensitivity and specificity. On one hand, sufficient centrifugation power is needed to accelerate the agglomeration. It drives the AuNPs and bound protein molecules to concentrate at the bottom of the tube, thus reducing the precipitation length from millimeters (solution height) to micrometers (cluster thickness) and, in the meanwhile, promotes the molecule interactions with the AuNP sensors even at ultralow target concentrations in a concentrated microenvironment at the bottom of the tube. As a result, the NasRED assay time is reduced to minutes while achieving high sensitivity. On the other hand, adequate vortex agitation is necessary to redisperse the AuNP precipitate caused by the non-specific binding after centrifugation. Vortex agitation disperses unbound AuNPs back into the supernatant, creating a clear distinction between the precipitated AuNP clusters (representing positive detection) and the free-floating AuNPs (for signal readout). Importantly, excessive vortex agitation can break down the AuNP clusters formed during the antigen–antibody reaction, thus negatively impacting sensitivity and specificity.

**Figure 2:**
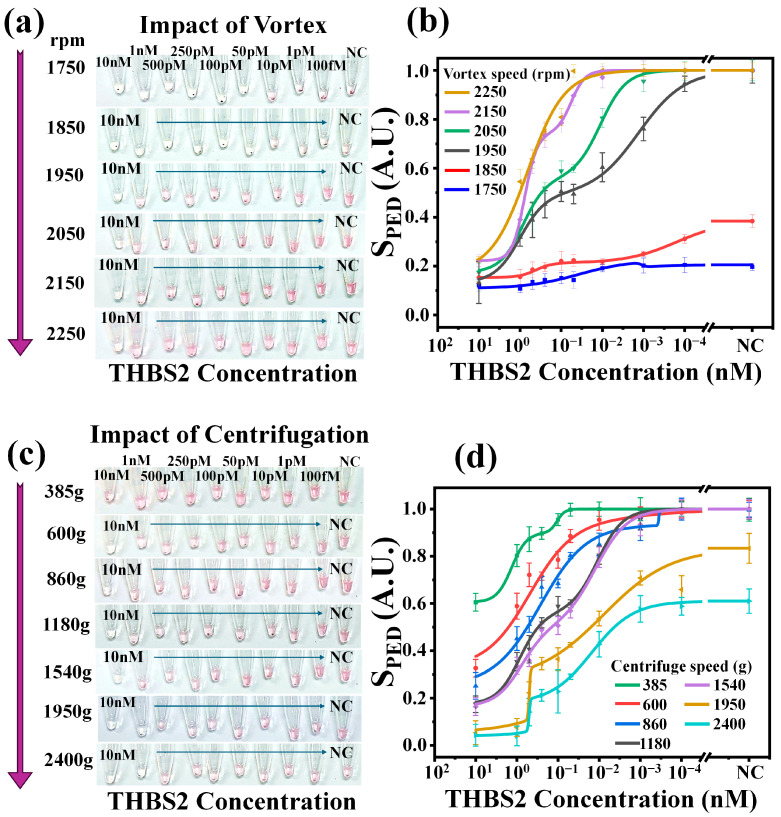
Sensing protocol optimization by analyzing the impact of active fluidic force. (**a**,**b**) Vortex agitation impact: (**a**) Optical images of the tubes immediately after the NasRED protocol with vortex speeds ranging from 1750 to 2250 rpm. (**b**) Sensing curves showing collected signals (S_PED_) plotted against different THBS2 concentrations on a logarithmic scale for different vortexing speeds. Here, S_PED_ is normalized between 1 (negative control, NC) and 0 (indicating positive sample). (**c**,**d**) Centrifugation impact: (**c**) Optical images of the tubes immediately after the readout with centrifugation speeds adjusted from 385× *g* to 2400× *g*. (**d**) Sensing curves showing collected signals S_PED_ plotted against different THBS2 concentrations on a logarithmic scale for different centrifugation speeds. Error bars represent the standard deviation across five independent readouts of the same tube in different orientations to account for tube variability.

To examine the impacts of centrifugation and vortex forces on the NasRED sensing performance, different concentrations of THBS2 (10 nM to 100 fM, and NC) in PBS media were mixed with functionalized AuNPs and tested under different parameters. For consistency, the sensing protocol was kept the same except for the vortex agitation speed (Figure 2a,b) and the centrifugation speed (Figure 2c,d). Clearly, low vortex speeds (1750 and 1850 rpm) did not create enough force to resuspend NC samples, which represented a blank solution without target proteins. The threshold value to redisperse the NC sample was found to be about 1950 rpm, but higher speeds could cause unintentional AuNP cluster breakdown and negatively impact the dynamic range (Figure 2b). Similarly, a large range of centrifugation conditions were tested, e.g., 400× *g* to 2400× *g* (2000 krpm to 4500 krpm). Clearly, AuNP agglomeration was excessive at higher centrifugation speeds (Figure 2c), leading to aggregate formation even for negative control samples that could not be broken up to resuspend after vortex agitation. These results (Figure 2d) indicated that higher centrifugation speeds (e.g., 1950× *g* and 2400× *g*) limited the maximum achievable PED signal values (to <0.8 and <0.6) and reduced the dynamic range compared with lower speeds. In comparison, lower centrifuge speeds (e.g., 385× *g* and 600× *g*) limited the minimum PED signals (to >0.6 and >~0.4) and also constrained the dynamic range of detection. These observations were used to empirically determine the optimal condition (1180× *g* and vortex at 1950 rpm) for the optimum dynamic range and LoDs.

### 3.3. THBS2 Detection

**Figure 3:**
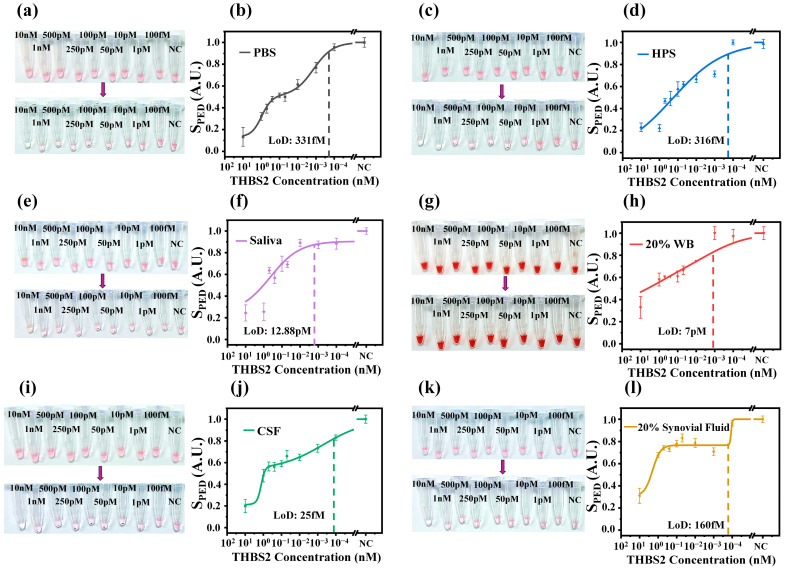
NasRED detection of THBS2 spiked in different biological matrices. (**a**,**b**) Detection in PBS: (**a**) optical images of sensing tubes before (top row) and after (bottom) the NasRED protocol, and (**b**) normalized sensing curve. (**c**,**d**) Detection in HPS: (**c**) optical images of sensing tubes, and (**d**) normalized sensing curve. (**e**,**f**) Detection in saliva. (**g**,**h**) Detection in 20% WB. (**i**,**j**) Detection in CSF. (**k**,**l**) Detection in 20% synovial fluid. All S_PED_ sensing curves are normalized between 1 (negative control, NC) and 0 (from positive sample, clear tube) and plotted against THBS2 concentrations on a logarithmic scale. Error bars represent the standard deviation across five independent readouts of the tube in different orientations to account for tube variability.

Our in-solution protein-sensing platform is adaptable to various targets in different media to detect THBS2 (Figure 3). In our previous study, we demonstrated the superior sensitivity of NasRED compared with ELISA by four orders of magnitude while providing accessibility, portability, and inexpensiveness [22]. THBS2, with a molecular weight of 129 kDa, has a neutralizing ability toward the anti-THBS2 antibody on the AuNPs, with the median effective dose (ED50) of 0.07–0.7 μg/mL and an LoD of 0.027 μg/mL (213 pM) in PBS (R&D Systems Inc.) [30]. The median reported THBS2 level in healthy patients’ serum was 5.8 ng/mL (45 pM) [9] and 24 ng/mL (186 pM) [18] measured on ELISA, while its cut-off values were found to be 40.9 ng/mL (317 pM) and 31.88 ng/mL (247 pM) for PDAC and NSCLC patients. The salivary level of THBS2 in healthy patient samples was 0.68 ± 0.73 ng/mL (5.2 ± 5.6 pM), which was significantly lower than that from OSCC patients at 12.90 ± 32.8 ng/mL (100 ± 254 pM) [11]. Additionally, THBS2 in the synovium is also an important endogenous regulator of angiogenesis and inflammation [17]. Based on these reports, THBS2 was spiked in PBS and biological media, including serum, WB, saliva, synovial fluid, and CSF, in the concentration range of 10 nM to 1 fM and tested on the NasRED platform to evaluate the feasibility of clinical cancer detection. Here, the WB matrix was diluted to 20% to minimize potential signal interference due to overlapping of optical absorption between hemoglobin and AuNPs. Also, synovial fluid has a high viscosity, which can affect the effectiveness of vortex agitation and, therefore, was diluted to 20%.

There were several notable observations. First, the microcentrifuge tubes in PBS (Figure 3a) displayed a color transition from transparent at 10 nM to reddish color at 100 fM and negative control, yielding an LoD of ~331 fM (42.7 pg/mL) (Figure 3b). The same was observed for other biological matrices (Figure 3c,e,i,k) except for 20% WB, for which the tube images could not properly illustrate this transition (Figure 3g) due to the red color from both the red blood cells and AuNPs. However, the PED signal measured from the light extinction changes at 532 nm (Figure 3h) was able to delineate the different THBS2 concentrations, suggesting the feasibility of direct detection from whole blood without blood cell separations to further simplify the clinical use. In terms of sensitivity, the readout signals (Figure 3d,f,h,j,l) proved the capability of THBS2 screening in different media with calculated LoDs as ~0.316 pM (40.8 pg/mL), 0.025 pM (3.2 pg/mL), 12.88 pM (1.6 ng/mL), 7 pM (903 pg/mL), and 0.16 pM (20.64 pg/mL) for HPS, CSF, saliva, 20% WB, and 20% synovial fluid, respectively. These measured values are comparable or better than those reported by ELISA assays. Lastly, considering the need for a very small amount of blood (6 µL of 20% whole blood or only ~1.2 µL), NasRED can be adopted to analyze minute amounts of samples from capillary blood, further minimizing the complexity of sample preparation.

**Figure 4:**
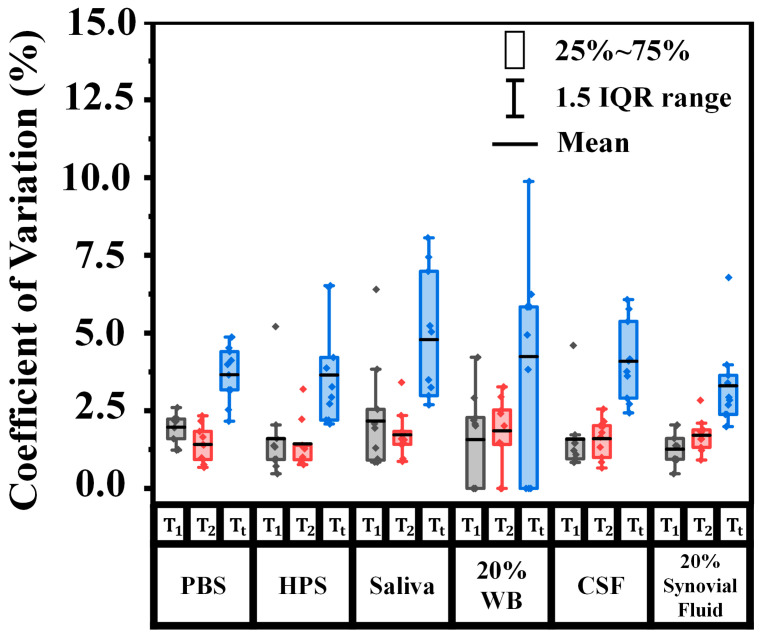
Measurement variation across tube orientations in biological matrices. The coefficient of variation (%) plotted against transmission signals before (gray boxes) and after (red boxes) protocol and delta transmission (blue boxes) in different biological matrices (PBS, HPS, saliva 20% WB, CSF, and 20% synovial fluid). Boxes represent the interquartile range (IQR), covering the 25% to 75% percentiles, while the vertical lines extend to 1.5 times the IQR. The mean values are indicated by the horizontal black lines.

The consistency of our device was validated by calculating the coefficient of variation from each step of measurement across different orientations of testing tubes in different biological matrices (Figure 4). Overall, the mean values of CV% of T_1_ and T_2_ (transmission signals before and after the sensing protocol) were both below 5% in all matrices, indicating minimal impacts on the signal intensities along different orientations. More specifically, the signal variations were smallest (<~3%) in PBS, HPS, CSF, and synovial fluid but slightly higher (3–5%) in saliva and 20% WB. This analysis indicated consistency across the tested biological fluids, which is important to ensuring biosensing signal reproducibility and precision. On the other hand, tests on saliva and 20% WB revealed both larger CV% values of T_t_ (8–10%) and worse LoDs (picomolars) than those on PBS, HPS, CSF, and synovial fluid (CV% < 7% and LoDs at femtomolars), suggesting a more significant biological matrix effect in saliva and blood.

### 3.4. Specificity Test

**Figure 5:**
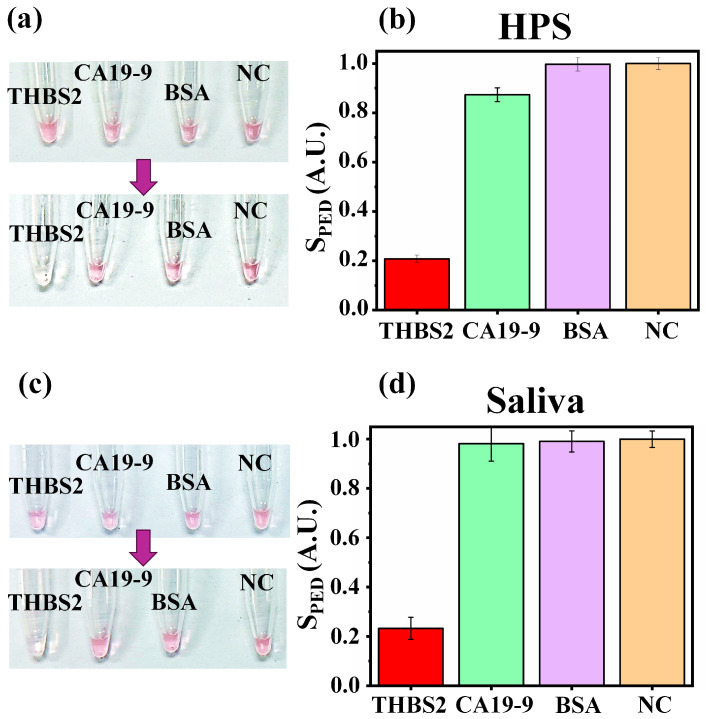
THBS2 specificity analysis in body fluids. (**a**,**b**) Specificity analysis in HPS: (**a**) Optical images of the tubes before (top row) and after (bottom row) the NasRED protocol, with THBS2 spiked at 10 nM concentration. (**b**) Bar charts representing normalized sensor signals (S_PED_) for THBS2 compared with CA19-9, BSA, and the negative control (NC). S_PED_ represents changes in optical extinction (see Section 2). (**c**,**d**) Specificity analysis in saliva.

To ensure that a diagnostic assay accurately identifies the target analyte and does not produce false positive results due to cross-reactivity with other substances, other proteins should be tested in the same conditions in similar sample media. To validate the specificity of NasRED for THBS2 detection, we included CA19-9 and BSA as comparison models in our analysis (Figure 5). These proteins were chosen due to their distinct biological relevance with the THBS2-specific antibody-functionalized AuNPs. CA19-9 serves as a clinically implemented tumor marker, particularly for pancreatic cancer. While CA19-9 and THBS2 can be elevated in certain cancers, they have different characteristics and roles and should be considered separate markers [4,10,18,20]. Protein blockers, such as BSA, are used to block non-specific bindings and stabilizers for the probes when performing the testing [31] and should not impact the signal. Here, HPS and saliva were chosen to assess the specificity of THBS2 sensing in biological matrices. Evidently, AuNP aggregation only happened in the tube samples with THBS2 (Figure 5a,c), and the tubes holding CA19-9, BSA, and blank samples (NC) did not produce noticeable color changes. Indeed, no significant PED signal was detected for these proteins (Figure 5b,d), confirming the absence of non-specific binding or cross-reactivity. These results underscored the high specificity of NasRED, ensuring that the observed signals were not negatively affected by the biological matrices or the presence of other protein markers.

## 4. Conclusions

Determining the THBS2 concentration in body fluids is a potentially important tool for cancer screening due to the overexpression of THBS2 in various cancers. Current diagnostic methods such as ELISA, PCR, and IHC are the main methods; however, they require lengthy assay times, bulky instruments, and professional operation, thus limiting their use in clinics and physician’s offices for rapid and convenient screening. Here, as a proof-of-concept demonstration we studied the application of NasRED as a plug-and-play, in-solution, POC platform to detect THBS2 for cancer prescreening. The NasRED platform was able to achieve a high sensitivity (LoD 331 fM in PBS) within 15 min, which is orders of magnitude better than that of ELISA (LoD 213 pM in PBS and typically >6 h turnaround time [30]). In this study, we optimized the NasRED assay protocol by analyzing the impacts of the active fluidic forces on the assay sensitivity in PBS and further demonstrated the feasibility of THBS2 detection in various biological matrices with high sensitivity (e.g., LoDs of 25 fM in CSF, 12.9 pM in saliva, and 7 pM in WB) and high specificity. The small amount of sample needed (<6 µL per test) also made it possible for NasRED to use less-invasive capillary blood and a minimal amount of CSF for routine screening, which would otherwise be much more challenging. The modularity of NasRED makes it easily engineerable in future work to detect other cancer biomarkers for cancer prescreening, prognostics, and diagnostics for a wide variety of cancer types, from oral cavity cancer and lymphoma to medulloblastoma, cervical cancer, PDAC, NSCLC, and melanoma.

## Data Availability

The original contributions presented in this study are included in the article. Further inquiries can be directed to the corresponding author.

## References

[1] Deng B., Liu X.-P., Wang X. (2021). Prognostic and Immunological Role of THBS2 in Colorectal cancer. BioMed Res. Int..

[2] Fei W., Chen L., Chen J., Shi Q., Zhang L., Liu S., Li L., Zheng L., Hu X. (2017). RBP4 and THBS2 are serum biomarkers for diagnosis of colorectal cancer. Oncotarget.

[3] Wang L., Feng L., Liu L., Han J., Zhang X., Li D., Liu J., Wang Y., Zuo J., Fan Z. (2023). Joint effect of THBS2 and VCAN accelerating the poor prognosis of gastric cancer. Aging.

[4] Li L., Dong J., Fu L., Xia X., Pan F., Ning Y. (2021). Clinical Value of Serum Thrombospondin-2 Combined with CA19-9 in Early Diagnosis of Gastric Cancer. J. Oncol..

[5] Chu X.-D., Lin Z.-B., Huang T., Ding H., Zhang Y.-R., Zhao Z., Huangfu S.-C., Qiu S.-H., Guo Y.-G., Pan J.-H. (2022). Thrombospondin-2 holds prognostic value and is associated with metastasis and the mismatch repair process in gastric cancer. BMC Cancer.

[6] Kim J., Bamlet W.R., Oberg A.L., Chaffee K.G., Donahue G., Cao X.-J., Chari S., Garcia B.A., Petersen G.M., Zaret K.S. (2017). Detection of early pancreatic ductal adenocarcinoma with thrombospondin-2 and CA19-9 blood markers. Sci. Transl. Med..

[7] Köbel M., Kang E., Lee S., Terzic T., Karnezis A.N., Ghatage P., Woo L., Lee C., Meagher N.S., Ramus S.J. (2024). Infiltrative pattern of invasion is independently associated with shorter survival and desmoplastic stroma markers FAP and THBS2 in mucinous ovarian carcinoma. Histopathology.

[8] Lin Y., Lin E., Li Y., Chen X., Chen M., Huang J., Guo W., Chen L., Wu L., Zhang X. (2022). Thrombospondin 2 is a Functional Predictive and Prognostic Biomarker for Triple-Negative Breast Cancer Patients with Neoadjuvant Chemotherapy. Pathol. Oncol. Res..

[9] Xiong L., Zhu C., Lu Y., Chen M., Li M. (2023). Serum THBS2 is a potential biomarker for the diagnosis of non-small cell lung cancer. J. Cancer Res. Clin. Oncol..

[10] Jiang Y.-M., Yu D.-L., Hou G.-X., Jiang J.-L., Zhou Q., Xu X.-F. (2019). Serum thrombospondin-2 is a candidate diagnosis biomarker for early non-small-cell lung cancer. Biosci. Rep..

[11] Hsu C.-W., Yu J.-S., Peng P.-H., Liu S.-C., Chang Y.-S., Chang K.-P., Wu C.-C. (2014). Secretome profiling of primary cells reveals that THBS2 is a salivary biomarker of oral cavity squamous cell carcinoma. J. Proteome Res..

[12] Wang X., Xu J., Hua F., Wang Y., Fang G., Zhang H., Wu X. (2023). MiR-214-3p suppresses cervical cancer cell metastasis by downregulating THBS2. Cell. Mol. Biol..

[13] Chang I.-W., Li C.-F., Lin V.C.-H., He H.-L., Liang P.-I., Wu W.-J., Li C.-C., Huang C.-N. (2016). Prognostic impact of thrombospodin-2 (THBS2) overexpression on patients with urothelial carcinomas of upper urinary tracts and bladders. J. Cancer.

[14] Kimura T., Iwadare T., Wakabayashi S., Kuldeep S., Nakajima T., Yamazaki T., Aomura D., Zafar H., Iwaya M., Joshita S. (2024). Thrombospondin 2 is a key determinant of fibrogenesis in non-alcoholic fatty liver disease. Liver Int..

[15] Liu Q.-H., Ma L.-S. (2018). THBS2 is a valuable prognosis biomarker in UM. Eur. Rev. Med. Pharmacol. Sci..

[16] He Z., Lin J., Chen C., Chen Y., Yang S., Cai X., He Y., Liu S. (2022). Identification of BGN and THBS2 as metastasis-specific biomarkers and poor survival key regulators in human colon cancer by integrated analysis. Clin. Transl. Med..

[17] Park Y.W., Kang Y.M., Butterfield J., Detmar M., Goronzy J.J., Weyand C.M. (2004). Thrombospondin 2 functions as an endogenous regulator of angiogenesis and inflammation in rheumatoid arthritis. Am. J. Pathol..

[18] Le Large T.Y., Meijer L.L., Paleckyte R., Boyd L.N., Kok B., Wurdinger T., Schelfhorst T., Piersma S.R., Pham T.V., van Grieken N.C. (2020). Combined Expression of Plasma Thrombospondin-2 and CA19-9 for Diagnosis of Pancreatic Cancer and Distal Cholangiocarcinoma: A Proteome Approach. Oncologist.

[19] Hirose Y., Chiba K., Karasugi T., Nakajima M., Kawaguchi Y., Mikami Y., Furuichi T., Mio F., Miyake A., Miyamoto T. (2008). A Functional Polymorphism in THBS2 that Affects Alternative Splicing and MMP Binding Is Associated with Lumbar-Disc Herniation. Am. J. Hum. Genet..

[20] Nan P., Dong X., Bai X., Lu H., Liu F., Sun Y., Zhao X. (2022). Tumor-stroma TGF-β1-THBS2 feedback circuit drives pancreatic ductal adenocarcinoma progression via integrin αvβ3/CD36-mediated activation of the MAPK pathway. Cancer Lett..

[21] Mirjalili S., Ikbal M.A., Hou C.-W., Mohammadi M.K., Choi Y., Kelbauskas L., VanBlargan L.A., Hogue B.G., Murugan V., Diamond M.S. (2024). Nanoparticle-Supported, Rapid, Digital Quantification of Neutralizing Antibodies Against SARS-CoV-2 Variants. bioRxiv.

[22] Choi Y., Mirjalili S., Ikbal M.D.A., McClure S., Clemens S., Solano J., Heggland J., Zuo J., Wang C. (2024). Nanoparticle-Supported, Rapid, and Electronic Detection of SARS-CoV-2 Antibodies and Antigens at Attomolar Level. bioRxiv.

[23] Ikbal M.D.A., Kang S., Chen X., Gu L., Wang C. (2023). Picomolar-Level Sensing of Cannabidiol by Metal Nanoparticles Functionalized with Chemically Induced Dimerization Binders. ACS Sensors.

[24] Chen X., Kang S., Ikbal A., Zhao Z., Pan Y., Zuo J., Gu L., Wang C. (2022). Synthetic nanobody-functionalized nanoparticles for accelerated development of rapid, accessible detection of viral antigens. Biosens. Bioelectron..

[25] Haiss W., Thanh N.T.K., Aveyard J., Fernig D.G. (2007). Determination of size and concentration of gold nanoparticles from UV-Vis spectra. Anal. Chem..

[26] Armbruster D.A., Pry T. (2008). Limit of Blank, Limit of Detection and Limit of Quantitation. Clin. Biochem. Rev..

[27] Deng L., Kitova E.N., Klassen J.S. (2013). Dissociation kinetics of the streptavidin-biotin interaction measured using direct electrospray ionization mass spectrometry analysis. J. Am. Soc. Mass Spectrom..

[28] Levenberg D.R., Varon E., Indech G., Ben Uliel T., Geri L., Sharoni A., Shefi O. (2023). A streptavidin–biotin system combined with magnetic actuators for remote neuronal guidance. J. Biol. Eng..

[29] Chivers C.E., Koner A.L., Lowe E.D., Howarth M. (2011). How the biotin–streptavidin interaction was made even stronger: Investigation via crystallography and a chimaeric tetramer. Biochem. J..

[30] Recombinant Human Thrombospondin-2 Protein, CF. https://www.rndsystems.com/products/recombinant-human-thrombospondin-2-protein-cf_1635-t2.

[31] Sun Y.-S., Zhu X. (2016). Characterization of Bovine Serum Albumin Blocking Efficiency on Epoxy-Functionalized Substrates for Microarray Applications. J. Lab. Autom..

